# Severe Dupilumab-Associated Psoriasis Flare After Switching From Ustekinumab for Biopsy-Proven Eczematous Dermatitis: A Case Report

**DOI:** 10.7759/cureus.114199

**Published:** 2026-08-09

**Authors:** Doaa AlAwadhi, Reem AlQusaimi, Fawziah AlRujaib, Fahad AlSharhan, Abdullah AlShatti, Bader Alflaij, Dana Alobaid, Suad Alassaf, Fatemah Abdulrahman, Munirah F AlEnezi

**Affiliations:** 1 Dermatology, Abdulkareem AlSaeed Dermatology Center, Kuwait City, KWT; 2 Medicine, Sheikh Jaber Al-Ahmad Al-Sabah Hospital, Kuwait City, KWT; 3 Medicine, Kuwait University, Kuwait City, KWT; 4 Medicine, Mirza Hassan Al Ahqaqee Clinic, Kuwait City, KWT; 5 Dermatology, Mussaaed Al-Saleh Health Center, Kuwait City, KWT; 6 Dermatology, Al Jahra Hospital, Kuwait City, KWT

**Keywords:** atopic dermatitis, biologic therapy, dupilumab, paradoxical psoriasis, ustekinumab

## Abstract

Dupilumab is an effective treatment for moderate-to-severe atopic dermatitis but has been increasingly associated with paradoxical psoriasis and psoriasiform manifestations. Severe exacerbation of pre-existing psoriasis following dupilumab therapy remains uncommon and presents a therapeutic challenge.

We report a patient with well-controlled chronic plaque psoriasis who developed eczematous dermatitis with histopathological findings consistent with the diagnosis. Although dupilumab effectively controlled the eczema, the patient subsequently developed a severe generalized psoriasis flare after switching from ustekinumab. Clinical improvement was achieved after restarting ustekinumab and methotrexate.

This case highlights the potential for dupilumab to trigger severe paradoxical psoriasis in patients with pre-existing psoriatic disease. Clinicians should remain vigilant when prescribing dupilumab in this population, and early recognition with prompt modification of therapy may lead to favorable clinical outcomes.

## Introduction

Dupilumab is a monoclonal antibody targeting the interleukin (IL)-4 receptor α subunit, thereby inhibiting IL-4 and IL-13 signaling, and is an effective treatment for moderate-to-severe atopic dermatitis. Although generally well tolerated, dupilumab has been increasingly associated with paradoxical psoriasis and psoriasiform manifestations (DAPs/PsM), an uncommon adverse event thought to result from immune dysregulation and a shift toward T helper (Th)1/Th17-mediated inflammation. In simple terms, atopic dermatitis is predominantly driven by Th2 immune responses, whereas psoriasis is primarily driven by Th1- and Th17-mediated inflammation. Altering this immune balance may therefore predispose susceptible individuals to the development or exacerbation of psoriasis [1]. Importantly, psoriasis developing after initiation of dupilumab should be distinguished from recurrence following withdrawal of an effective psoriasis-directed therapy, as both may present with similar clinical manifestations. Furthermore, the coexistence of psoriasis and eczematous dermatitis presents a therapeutic challenge because treatment directed toward one inflammatory pathway may inadvertently exacerbate the other.

DAPs/PsM include de novo psoriasis, exacerbation of pre-existing psoriasis, and psoriasiform dermatitis. Chronic plaque psoriasis is the most commonly reported subtype, although guttate, pustular, erythrodermic, inverse, and sebopsoriasis have also been described. The reported incidence ranges from approximately 1.8% to 3.3%, with most cases developing within the first year of treatment [2].

We report a rare case of severe paradoxical exacerbation of chronic plaque psoriasis following initiation of dupilumab for eczematous dermatitis with histopathological findings consistent with an eczematous reaction pattern. Despite marked improvement of eczema, the patient developed a generalized psoriasis flare requiring hospitalization, which was successfully managed with discontinuation of dupilumab and reintroduction of ustekinumab in combination with methotrexate (MTX). This case highlights the importance of recognizing this uncommon adverse effect, particularly in patients with a history of psoriasis.

## Case presentation

An 84-year-old Kuwaiti man with a medical history significant for well-controlled hypertension and type 2 diabetes mellitus was diagnosed with chronic plaque psoriasis in 2015. There was no personal or family history of atopic disease or psoriasis. He initially received topical therapies with inadequate disease control and was subsequently started on subcutaneous MTX. Baseline laboratory investigations demonstrated normal inflammatory markers and liver function, with mild macrocytosis, a slightly reduced red blood cell count, borderline renal function, hypercholesterolemia, and hyperuricemia (Table 1). The MTX dose was adjusted between 7.5 and 15 mg weekly according to disease activity, achieving satisfactory disease control with regular laboratory monitoring and folic acid supplementation. In 2018, the patient experienced a mild flare involving the elbows and knees, which responded to a temporary increase in MTX to 12.5 mg weekly before returning to maintenance therapy (Figure 1). Follow-up laboratory monitoring remained stable, showing only mild elevation of creatinine, persistent hyperuricemia, and normal liver function tests.

**Table 1:** Baseline laboratory investigations before methotrexate initiation (January 12, 2015). Baseline laboratory investigations demonstrated mild macrocytosis, borderline renal function, hypercholesterolemia, and hyperuricemia, while inflammatory markers and liver function tests were within normal limits.

Test	Value	Reference range (unit)
White blood cell count	8.4	4-10 (10^9^/L)
Red blood cell count	3.7	3.8-4.8 (10^12^/L)
Hemoglobin	143	120-150 (g/L)
Platelet count	204	150-410 (10^9^/L)
Mean corpuscular volume	103	80-100 (fL)
Creatinine	104	49-90 (umol/L)
Sodium	140	136-144 (mmol/L)
Potassium	4.63	3.6-5.1 (mmol/L)
Albumin	38	35-52 (g/L)
Total bilirubin	20	5-21 (umol/L)
Alanine aminotransferase	17	3-35 (U/L)
Aspartate aminotransferase	24	3-35 (U/L)
Total cholesterol	5.7	3-5.2 (mmol/L)
Triglyceride	1.93	0.4-1.75 (mmol/L)
Erythrocyte sedimentation rate	8	0-20 (mm/hr)
Uric acid	496	210-420 (umol/L)

**Figure 1:**
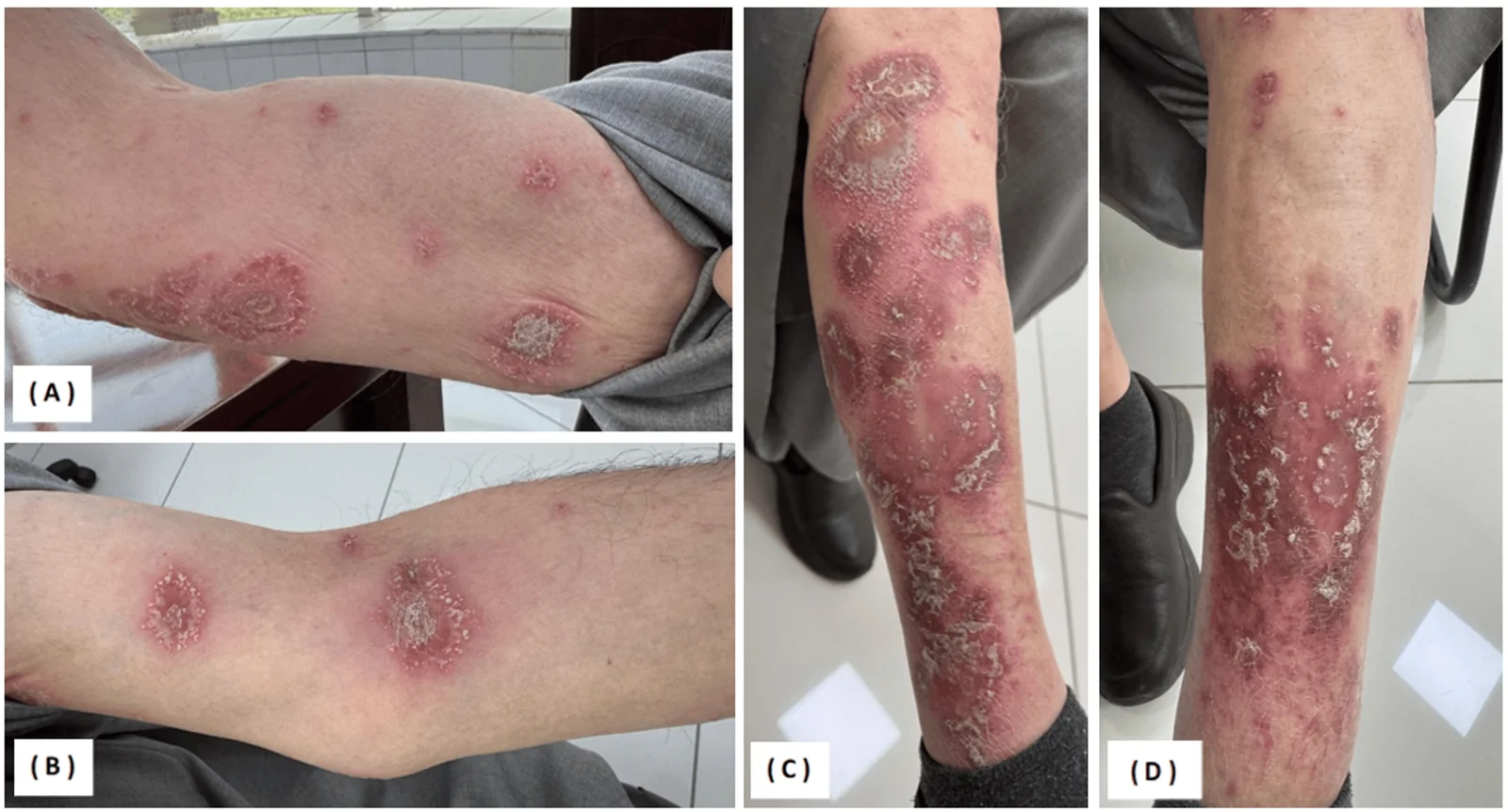
Mild psoriasis flare involving the upper and lower extremities during methotrexate therapy (2018). (A, B) Clinical photographs of the upper extremity demonstrating well-demarcated, erythematous, scaly plaques over the elbow. (C, D) Clinical photographs of the lower extremities showing similar psoriatic plaques over the legs. The flare responded to a temporary increase in methotrexate from 7.5 mg to 12.5 mg weekly, with subsequent restoration of disease control.

During evaluation for biologic therapy in 2021, routine investigations revealed mild normocytic anemia, persistent macrocytosis, mildly impaired renal function, and persistent hyperuricemia, while liver function tests remained within normal limits (Table 2). Screening for hepatitis B, hepatitis C, and human immunodeficiency virus was negative. Antinuclear antibody testing demonstrated a low-titer speckled pattern (1:80), and an interferon-gamma release assay (T-SPOT.TB) was positive, consistent with latent tuberculosis infection. Consequently, MTX was discontinued, and the patient completed treatment with isoniazid and rifampicin before initiation of biologic therapy (Table 3).

**Table 2:** Pre-biologic laboratory investigations before ustekinumab initiation (May 2021). Routine laboratory investigations demonstrated mild anemia, persistent macrocytosis, mildly impaired renal function, and hyperuricemia, while liver function tests remained within normal limits.

Test	Value	Reference range (unit)
White blood cell count	5.3	4-10 (10^9^/L)
Red blood cell count	3.62	3.8-4.8 (10^12^/L)
Hemoglobin	128	120-150 (g/L)
Platelet count	151	150-410 (10^9^/L)
Mean corpuscular volume	106	80-100 (fL)
Creatinine	124	49-90 (umol/L)
Albumin	41	35-52 (g/L)
Total bilirubin	6.5	5-21 (umol/L)
Alanine aminotransferase	13	3-35 (U/L)
Aspartate aminotransferase	25	3-35 (U/L)
Total cholesterol	5.4	3-5.2 (mmol/L)
Uric acid	463	210-420 (umol/L)

**Table 3:** Pre-biologic infectious disease and tuberculosis screening before ustekinumab initiation (May 2021). Screening for hepatitis B, hepatitis C, and HIV was negative. Tuberculosis screening demonstrated a reactive T-SPOT.TB result, consistent with latent tuberculosis infection, necessitating completion of antituberculous therapy before initiation of biologic treatment. HBsAg, hepatitis B surface antigen; anti-HCV, hepatitis C virus antibody; HIV Ag/Ab, human immunodeficiency virus antigen/antibody; T-SPOT.TB, T-cell spot test for tuberculosis

Virology	
Test	Results
HBs Ag in blood	Non-reactive
Anti-HCV in blood	Non-reactive
HIV Ag/Ab in blood	Non-reactive
Tuberculosis screening	
Test	Results
T-SPOT.TB	Reactive

In June 2022, the patient weighed 82 kg and was therefore initiated on the standard ustekinumab regimen of 45 mg administered subcutaneously according to the recommended induction schedule (weeks 0 and 4, followed by every 12 weeks). The patient achieved complete clearance of psoriatic lesions by October 2022 and maintained remission throughout 2023 and most of 2024. A mild recurrence of lesions occurred after missing one scheduled ustekinumab dose but resolved following treatment resumption.

In January 2025, while his psoriasis remained in complete clinical remission with no active psoriatic plaques on examination during ustekinumab therapy, the patient developed a generalized, intensely pruritic eczematous eruption predominantly affecting the face and dorsal hands, associated with lichenification, excoriations, intermittent oozing, and sleep disturbance. The eruption responded only transiently to potent topical corticosteroids, topical tacrolimus, emollients, antihistamines, and a short course of oral prednisolone. The differential diagnosis included allergic contact dermatitis, irritant contact dermatitis, drug-induced eczematous dermatitis, chronic actinic dermatitis, and eczematized psoriasis. Skin biopsy from the dorsal hand demonstrated focal parakeratosis, mild acanthosis, epidermal spongiosis, and a superficial perivascular lymphocytic infiltrate, with findings consistent with eczematous dermatitis (Figure 2). In conjunction with the clinical presentation, these findings supported the diagnosis of eczematous dermatitis.

**Figure 2:**
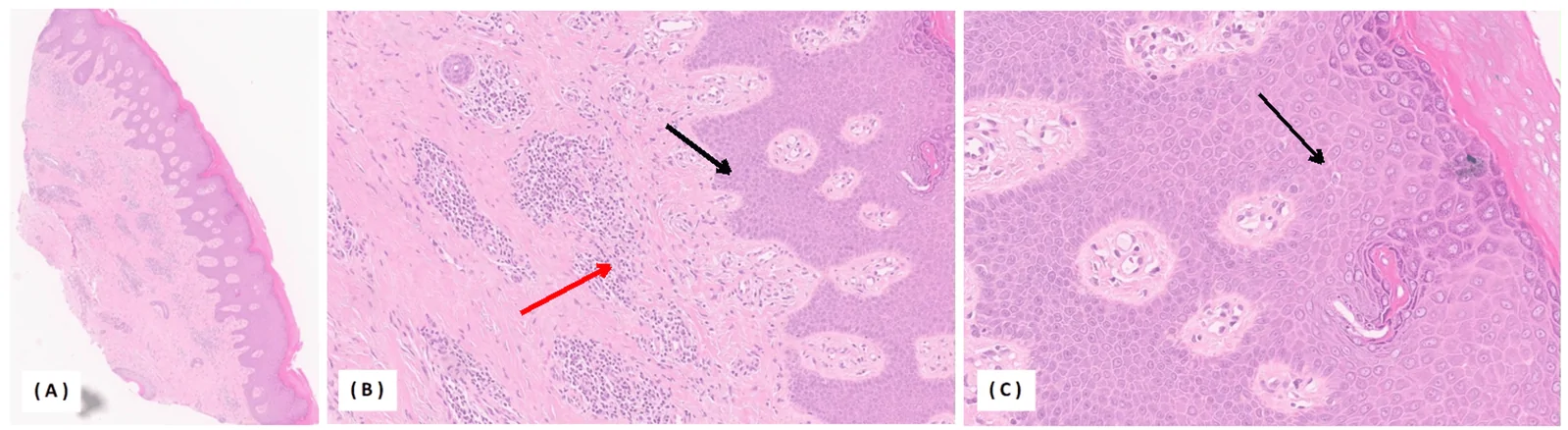
Histopathological findings consistent with eczematous dermatitis. Skin biopsy obtained from the dorsal hand (hematoxylin and eosin stain). (A) Low-power view demonstrating the overall epidermal and dermal architecture. (B) Medium-power view showing mild epidermal acanthosis with spongiosis (black arrow) and a superficial perivascular lymphocytic inflammatory infiltrate (red arrow) within the papillary/superficial dermis. (C) High-power view highlighting epidermal spongiosis (black arrow). These findings are consistent with eczematous dermatitis.

As the eczematous eruption remained refractory to conventional therapy despite complete control of psoriasis on ustekinumab, the decision was made to discontinue ustekinumab and initiate dupilumab. Dupilumab was initiated on January 22, 2025, with a 600 mg loading dose, followed by 300 mg every two weeks. The patient received maintenance doses on February 5, February 19, and March 5, 2025, resulting in near-complete resolution of the eczematous eruption while continuing emollients, topical corticosteroids, and topical tacrolimus. The patient was subsequently lost to follow-up until May 7, 2025, at which time continuation of dupilumab 300 mg every two weeks was planned.

Several weeks after initiation of dupilumab, while the eczema remained well controlled, the patient developed recurrent psoriatic lesions that rapidly progressed to a severe generalized flare involving the upper and lower extremities and genital region. This was accompanied by fever, chills, malaise, and fatigue. Dupilumab was subsequently discontinued, and oral MTX (7.5 mg weekly) with folic acid supplementation was initiated. Despite treatment, psoriasis progressed to generalized disease with erythroderma and pustular lesions involving more than 30% of the body surface area, necessitating hospitalization. Admission laboratory investigations demonstrated mild thrombocytosis with mildly elevated serum creatinine and uric acid levels; the remaining laboratory parameters were unremarkable. Infectious evaluation was negative. Given the patient's previous excellent response to ustekinumab, ustekinumab (45 mg) was reintroduced in combination with MTX.

Following discharge in March 2026, the patient demonstrated marked clinical improvement, with complete resolution of pustular lesions and substantial improvement of the generalized erythroderma. Residual psoriatic plaques showed significantly reduced erythema, scaling, and pruritus. MTX was increased to 10 mg weekly and subsequently to 12.5 mg weekly because of persistent disease activity. Laboratory investigations demonstrated macrocytosis without anemia, elevated serum urea and creatinine, and an estimated glomerular filtration rate (eGFR) of 44 mL/min/1.73 m², compared with 55 mL/min/1.73 m² in 2023. Nephrology assessment confirmed stable mild chronic kidney disease, and continuation of low-dose MTX with close monitoring of renal function, liver function tests, and complete blood counts was considered appropriate. At review four weeks after restarting ustekinumab, the Psoriasis Area and Severity Index (PASI) had improved to approximately 7 [3].

At subsequent follow-up visits in April and May 2026, the patient demonstrated continued clinical improvement, with a PASI of approximately 7, accompanied by marked reductions in erythema, scaling, and plaque thickness while receiving ustekinumab every 12 weeks and MTX 12.5 mg weekly (Figure 3). Repeat laboratory investigations showed persistent macrocytosis without anemia, while white blood cell count, lymphocyte count, and liver function tests remained within normal limits. In June 2026, psoriasis remained well controlled; however, the patient experienced an isolated flare of eczematous dermatitis involving the right cheek, presenting with edema, erythema, intense pruritus, and oozing, which was managed with intensified topical therapy while systemic psoriasis treatment was continued (Table 4).

**Figure 3:**
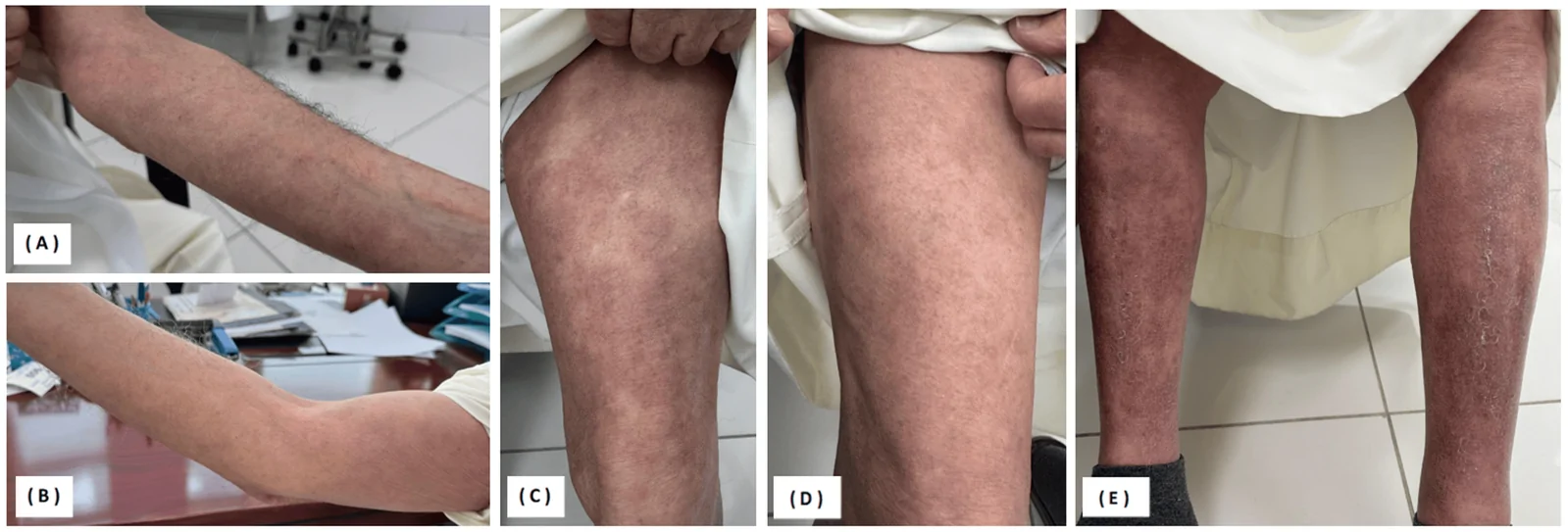
Clinical improvement following reintroduction of ustekinumab and methotrexate. (A, B) Upper extremities demonstrating marked reductions in erythema, scaling, and plaque thickness. (C-E) Lower extremities showing substantial clinical improvement following treatment with ustekinumab every 12 weeks and methotrexate 12.5 mg weekly. These photographs were obtained during follow-up visits in April and May 2026 and illustrate post-treatment improvement; photographs of the acute generalized psoriasis flare were not available.

**Table 4:** Chronological timeline of the patient's clinical course, treatment, and clinical outcomes. The table summarizes the patient's disease course from initial psoriasis diagnosis through biologic therapy, development of eczematous dermatitis, paradoxical psoriasis flare following dupilumab, subsequent management, and clinical outcomes. BSA, body surface area; PASI, Psoriasis Area and Severity Index

Date	Clinical event	Management	Outcome
2015	Diagnosed with chronic plaque psoriasis.	Started methotrexate.	Good disease control achieved.
2018	Mild psoriasis flare.	Increased methotrexate dose.	Flare resolved.
2021	Pre-biologic screening revealed latent tuberculosis.	Completed anti-tuberculosis treatment.	Cleared for biologic therapy.
June 2022	Initiated ustekinumab.	Standard induction and maintenance regimen.	Complete psoriasis remission by October 2022.
2024	Mild psoriasis relapse after missed ustekinumab dose.	Resumed ustekinumab.	Disease regained control.
January 2025	Developed severe generalized eczematous dermatitis.	Switched to dupilumab.	Eczema markedly improved.
Late 2025/early 2026	Severe psoriasis flare while on dupilumab.	Dupilumab discontinued; methotrexate restarted.	Psoriasis progressed despite treatment.
March 2026	Hospitalized for severe generalized psoriasis (>30% BSA).	Restarted ustekinumab with methotrexate.	Marked clinical improvement.
April-May 2026	Follow-up.	Continued combination therapy.	Achieved PASI 75 response.
June 2026	Localized eczema flare with controlled psoriasis.	Intensified topical therapy.	Psoriasis remained controlled.

## Discussion

DAPs/PsM are increasingly recognized as paradoxical inflammatory reactions that occur following initiation of dupilumab therapy. Although dupilumab effectively inhibits IL-4 and IL-13 signaling through blockade of the IL-4 receptor α subunit and is highly effective in treating atopic dermatitis, suppression of the Th2 pathway may result in a relative predominance of Th1/Th17-mediated inflammation, predisposing susceptible individuals to the development or exacerbation of psoriasis [1].

DAPs/PsM may present as de novo psoriasis in patients without a previous history of psoriasis or as an exacerbation of pre-existing psoriasis. The latter appears less common but is of particular clinical importance because patients with a known history of psoriasis may experience severe disease flares despite excellent control of their atopic dermatitis. In addition to classic psoriasis, dupilumab has also been associated with psoriasiform eruptions that demonstrate overlapping clinical and histopathological features of both psoriasis and eczema [4].

The most frequently reported clinical subtype is chronic plaque psoriasis, presenting as well-demarcated, erythematous plaques with adherent silvery scale. Less commonly, guttate, pustular, erythrodermic, inverse psoriasis, and sebopsoriasis have been described. Lesions may involve the trunk, extremities, scalp, face, genital region, and intertriginous areas. Histopathological examination generally demonstrates features consistent with psoriasis, including acanthosis, parakeratosis, elongation of rete ridges, and dilated dermal capillaries, although some cases exhibit concomitant spongiosis, reflecting an overlap between psoriatic and eczematous inflammation [5].

The onset of DAPs/PsM is variable, typically occurring several weeks to months after initiation of dupilumab therapy, although earlier and later presentations have been reported. Clinical severity ranges from localized plaques responsive to topical therapy to extensive generalized disease requiring discontinuation of dupilumab and transition to alternative systemic or biologic treatment [2].

Several risk factors for DAPs/PsM have been proposed, although the condition remains largely unpredictable. A personal or family history of psoriasis is considered the most important predisposing factor, suggesting that dupilumab may unmask latent psoriatic disease or precipitate relapse in genetically susceptible individuals. Nevertheless, paradoxical psoriasis has also been reported in patients without any previous history of psoriasis, indicating that additional genetic and immunologic mechanisms are likely involved. At present, no validated biomarkers are available to identify patients at increased risk before initiating dupilumab therapy [6].

Management of DAPs/PsM depends on disease severity and the degree of control of the underlying atopic dermatitis. Mild localized disease may respond to topical corticosteroids, vitamin D analogues, topical calcineurin inhibitors, or phototherapy while continuing dupilumab. In contrast, moderate-to-severe disease often requires discontinuation of dupilumab and initiation of systemic therapy, including MTX, cyclosporine, apremilast, or biologic agents targeting the IL-23/IL-17 pathway [7]. In the present case, dupilumab was selected because the patient's psoriasis was in complete clinical remission while the eczematous dermatitis remained refractory to conventional therapy despite optimized topical treatment and significantly impaired quality of life. Although DAPs/PsM are increasingly recognized, they remain relatively uncommon, and the anticipated therapeutic benefits of dupilumab were considered to outweigh the potential risk of paradoxical psoriasis at the time treatment was initiated.

One important limitation of the present case is the difficulty in establishing causality. The patient had previously experienced recurrence of psoriasis after missing a scheduled ustekinumab dose, suggesting that withdrawal of effective psoriasis therapy may have contributed to the subsequent flare. In addition, the contribution of the preceding short course of systemic corticosteroids cannot be completely excluded. Although withdrawal of ustekinumab represents an important competing explanation, the temporal sequence and clinical course observed in this case are consistent with previously reported cases of DAP and PsM. Nevertheless, the close temporal relationship between discontinuation of ustekinumab, initiation of dupilumab, and the development of extensive generalized psoriasis, together with the growing body of literature describing DAP and PsM, supports a possible association. As with many published case reports, a definitive causal relationship cannot be established.

## Conclusions

This case highlights the therapeutic challenge of managing patients with coexisting psoriasis and eczematous dermatitis, in whom treatment of one inflammatory condition may inadvertently be associated with worsening of the other. Although dupilumab achieved near-complete resolution of the eczematous dermatitis, the patient subsequently developed a severe generalized flare of previously well-controlled chronic plaque psoriasis following discontinuation of ustekinumab and initiation of dupilumab, ultimately requiring hospitalization. While the temporal relationship raises concern for a DAP flare, the contribution of ustekinumab withdrawal cannot be excluded. Reintroduction of ustekinumab in combination with MTX resulted in significant clinical improvement, suggesting that reinstatement of psoriasis-directed therapy may be an effective management strategy in similar cases. This case emphasizes the importance of carefully evaluating patients with a history of psoriasis before switching biologic therapies, closely monitoring for disease recurrence during treatment transitions, and promptly recognizing possible paradoxical or treatment-associated psoriasis flares to optimize patient outcomes.
